# Suicidal ideation among people with different gambling behaviour profiles: analysis of a longitudinal survey of people who gamble regularly in the UK

**DOI:** 10.1192/bjo.2025.10935

**Published:** 2026-01-15

**Authors:** Heather Wardle, Karen Wetherall, Jessica Wyllie, Sarah Tipping, Seonaid Cleare, Martin Jones, Sally McManus, Rory C. O’Connor

**Affiliations:** School of Social and Political Sciences, University of Glasgow, Glasgow, UK; Suicidal Behaviour Research Laboratory, School of Health and Wellbeing, University of Glasgow, Glasgow, UK; Independent; School of Health and Medical Sciences, City St George’s, University of London, London, UK

**Keywords:** Gambling, longitudinal, self-harm, suicide

## Abstract

**Background:**

People who gamble experience elevated rates of suicidal thoughts and behaviours. Longitudinal studies have been scarce, and none has focused on those who regularly gamble in the UK.

**Aims:**

To examine the relationship between specific products and locations of gambling activity (and their combinations) and risk of subsequent suicidal thoughts.

**Method:**

We analysed a UK longitudinal survey of 3927 adults (18 years old or over) who regularly bet on sports. Data were collected online between June and November 2020. Latent class analysis was used to identify groups of people with similar gambling profiles on the basis of 13 types of gambling activity. Weighted group characteristics are presented. Regression modelling was used to test associations between gambling groups and suicidal thoughts, adjusting for baseline characteristics.

**Results:**

Five distinct groups were identified. One group (5.6% of the sample) reported multiple types of both in-person and online gambling. This group was the most likely to use electronic gambling machines. After adjustment for baseline suicidal thoughts, this group had significantly higher odds of subsequent suicidal thoughts (adjusted odds ratio 3.42; 95% CI: 1.18–9.89) than other groups.

**Conclusions:**

Although many profiles of gambling activity present suicide risk, some types present greater risk. National Institute for Health and Care Excellence guidelines recommend enquiry in primary care settings about gambling behaviours. Our findings suggest that clinicians should consider asking questions on mode (online or in-person) and product (especially electronic gambling machines) to identify those at heightened risk of suicidal ideation. Gambling should also be considered routinely in psychosocial assessments across clinical settings and incorporated into suicide prevention campaigns.

Gambling can be a dominant factor in suicidality.^1^ Those with lived experience of gambling harms often highlight how suicidal ideation and attempts were a feature of their experience.^2^ In Great Britain, there are an estimated 117 to 496 gambling-related suicides per year^3^ and coronial investigations have recognised gambling as a factor for suicide in Britain and elsewhere,^4–6^ but major gaps in knowledge remain. In Britain, National Institute of Health and Care Excellence guidelines on the identification, assessment and management of gambling-related harms recommend that health and social care practitioners consider asking about gambling alongside behaviours such as smoking and alcohol consumption.^7^ These guidelines specifically recommend that professionals ask about gambling during presentations related to mental health, including suicidal thoughts and behaviours, using direct questions such as asking whether someone gambles or is worried about their own or someone else’s gambling. Identification of gambling as a dominant or complicating factor for an individual may improve their health outcomes through effective treatment and management of gambling alongside other clinical recommendations and care pathways. Although the overarching relationships of gambling disorder and experience of problem gambling with suicidality have been well documented,^8–14^ to inform better professional inquiry, there is an urgent need to understand whether certain types of gambling products or gambling modalities (i.e. online, offline) are more strongly associated with subsequent suicidal ideation than others. Further, as many people who gamble engage in more than one gambling format, there is a need to understand suicide risk in relation to specific clusters of behaviours. Understanding how risk may be heightened among people who engage in different types and combinations of gambling activities is essential to inform the implementation of effective identification and assessment of harms. The current study aimed to fill this gap.

## Current study

Using longitudinal data collected from a large sample of people in the UK who gambled regularly, we assessed the association between different patterns of gambling and subsequent suicidal ideation. To our knowledge, this is the only longitudinal sample of people who have regularly gambled currently available in the UK. Its large sample size provides an opportunity to identify and examine different patterns of gambling behaviours in those who gamble regularly and thus to explore the associations of these patterns with suicidal ideation. We also explored relationships with suicide attempts as supplementary analyses (which were limited by small sample sizes). The aims of the study were to:

- identify a typology comprising groups of people with similar profiles of gambling activities in terms of types, products and modalities;
- describe the sociodemographic, economic and other characteristics of those in each group;
- compare each group’s likelihood of subsequent suicidal ideation and attempts, adjusting for baseline characteristics and experiences.

## Method

### Design and sample

Data were taken from a longitudinal survey of adults in the UK who regularly bet on sports (18+ years old; bet on sports at least monthly),^15^ originally designed to monitor the impact of the COVID-19 pandemic. Participants completed an online survey three times between June 2020 and April 2021. This study used data collected in waves 1 and 2 (wave 1, *n* = 3927: June 2020; wave 2, *n* = 3195: November 2020). These waves were chosen as wave 1 specifically captured gambling behaviours in the 3 months before the onset of the COVID-19 pandemic, and wave 2 collected data with reference to behaviours captured between August and October 2020, when all gambling opportunities had recommenced after the first national lockdown (March–June 2020). Wave 3 collected information on behaviours during a further national lockdown (January–March 2021), when land-based gambling venues were closed, substantially affecting gambling opportunities and behaviours during this time. Wave 3 was not included here as in-person gambling was circumscribed, rendering comparison with waves 1 and 2 problematic.

Participants were recruited by YouGov from their non-probabilistic online panel of more than one million members living in the UK. Participants were eligible if they: had gambled at least monthly on sports (including horse and dog races) between February 2019 and February 2020 (identified by data held by YouGov); were aged 18 years or over; and had not taken part in another YouGov study on gambling in the past year. In each wave, participants were contacted by YouGov via direct e-mail invitations. Participants received YouGov points (equivalent to £0.50, redeemable for vouchers) as remuneration. Up to three reminders were sent (further information published elsewhere^15,16^). Written consent was indicated through formal and expressed consent given by participants at the outset of the survey. This was obtained after participants had been presented with full information on the purpose of the survey, who was running it and why, confirmation that it was voluntary and what would happen to data provided. Written consent to be part of the YouGov panel and to take part in studies such as these is maintained by the YouGov team.

The profile of those participating was compared with that of all eligible YouGov participants. Cross-sectional and longitudinal weights were provided to match the achieved sample to the population profile (age, gender, ethnicity, number of gambling activities, frequency of gambling, and Problem Gambling Severity Index (PGSI) score) of those who gamble regularly according to the whole YouGov database and to develop weights to account for attrition.

Our analysis plans were reviewed by co-author M.J., who has lived experience of gambling harms, and by other people with lived experience of harms. M.J. waived remuneration for his time; other people with lived experience were paid for their contributions to the project.

The authors assert that all procedures contributing to this work comply with the ethical standards of the relevant national and institutional committees on human experimentation and with the Helsinki Declaration of 1975, as revised in 2013. All procedures were approved by the University of Glasgow College of Social Sciences Ethics Committee (ref no. 400230043).

### Measures

#### Gambling

The exposure was gambling activity. Participants were asked how often they had bet on 23 different gambling activities in the 3 months before the onset of the COVID-19 pandemic (December 2019 to February 2020) on an eight-point scale (from ‘several times a day’ to ‘less than once a month’). A binary variable (no/yes) for each activity indicated engagement once a fortnight or more. Owing to low prevalence, some activities (including esports betting and other sports betting) were grouped, yielding 13 gambling variables. These were: horses online betting, sports online betting, casino and/or poker online, slots online, bingo online, horses in-person, sports in-person, casino and/or poker in-person, slots in-person, bingo in-person, lottery, scratchcards and football pools (Supplementary Table 1 available at https://doi.org/10.1192/bjo.2025.10935). Owing to low numbers, betting on ‘other, non-sporting, events’ was excluded.

Participants completed the nine-item PGSI in both waves. The PGSI asked about gambling in the prior 3 months, and items were scored on a four-point scale (0 = ‘never’ to 3 = ‘almost always’), and a composite score (0 to 27) was computed (Cronbach’s *α* = 0.948 (wave 1)). Wave 1 data were used. PGSI scores were grouped into: 0–2 and 3+ (representing moderate risk or problem gambling).^17^

For both waves, participants were asked whether gambling had caused difficulties with finances, relationships, or mental and/or physical health in the past 3 months. Response options were ‘No difficulties’, ‘Yes, some difficulties’ and ‘Yes, many difficulties’, dichotomised as no/yes. For each gambling activity undertaken (except lotteries and scratchcards), participants were asked how much time they spent on that activity. Response options were presented on an eight-point scale ranging from ‘Less than 30 min per day’ to ‘8 h or more a day’. From this, an overall time spent gambling variable was derived with three categories: less than 30 min per day, 30 min to 3 h per day, and 3 h or more per day. For each gambling activity undertaken, participants were asked how much money they had spent. This was an open text question. Total gambling spend was derived, banded into three categories: £0–50, £51–250 and £251+.

#### Suicidal ideation

The primary outcome for the study was suicidal thoughts in the past 3 months. Questions about suicide attempts were also asked but because of small bases these are presented in the supplementary analysis only (Supplementary Table 3). Questions about suicidality were adapted from the Adult Psychiatric Morbidity Survey^18^ and asked ‘in the last 3 months, have you ever thought of taking your life, even if you would not actually do it?’ and ‘in the last 3 months, have you ever made an attempt to take your life, by taking an overdose of tablets or in some other way?’. Response options were ‘No’, ‘Yes’ or ‘Prefer not say’, with the latter treated as missing data. At wave 2, 102 (3.2%) and 152 participants (4.8%), respectively, did not answer the suicidal ideation and suicide attempt questions.

#### Well-being

The seven-item Short Warwick-Edinburgh Mental Wellbeing Scale was used to measure general well-being in the past 2 weeks.^19^ Total raw scores were transformed using the Short Warwick-Edinburgh Mental Wellbeing Scale conversion table. In the current study, scores were split into low (7–20), moderate (21–27) and high (28+) well-being. The composite score for wave 1 had acceptable internal consistency (Cronbach’s *α* = 0.85).

#### Alcohol consumption

Risky alcohol consumption was identified using the three-item Alcohol Use Disorders Identification Test-C.^20^ The questions were scored from 0 to 12. A score of 5 or more was considered to indicate a positive screen for alcohol consumption of increased risk of being hazardous to health.

#### Sociodemographic measures

Sociodemographic items included: gender (male, female), age (18–34, 35–54, 55+ years), ethnicity (White, Asian/Black/other), employment (employed, retired, unemployed/other), education (degree and above, school-level/other/none), occupational grade (managerial/professional: ABC1, routine/manual: C2DE), tenure (home-owner, renting/social housing), marital status (married/cohabiting, not) and longstanding limiting health problems or disability (yes, no).

### Statistical analysis

#### Latent class analysis (LCA)

LCA was used to identify groups of people with common patterns of gambling behaviours across 13 gambling formats in the 3 months before the COVID-19 pandemic. The aim of LCA is to enable identification of previously unknown subgroups of individuals within the data,^21^ in this case, gambling behaviour subgroups. The resulting LCA groups were used to identify which combinations of gambling activities posed the highest risk of suicidal thoughts and attempts, cross-sectionally and longitudinally. Mplus version 8.6 for Windows (Muthén & Muthén, Los Angeles, USA; https://www.statmodel.com/index.shtml) was used, and we fitted one to six latent class models to determine the optimal number of classes.

The final LCA model selection was based on fit statistics and interpretability, recognising that model fit statistics alone are insufficient as the resulting model needs to be interpretable and applicable^22^ (see Supplementary Fig. 1 and Supplementary Table 2 for further details of fit statistics).

#### Descriptive analyses

Bivariate analysis was used to examine the overall demographic, socioeconomic and gambling behaviours for (a) the total sample and (b) each latent class group, calculating Pearson’s chi-squared to test differences between groups. Analysis of variance was used for continuous variables. All variables were measured at wave 1 and weighted using wave 1 cross-sectional weights to adjust for non-response. Missing data were minimal and omitted from analyses.

#### Multivariable analyses

To test whether any of the LCA groups predicted suicidal ideation at wave 2, a binary logistic regression model was used. The outcome variable for the model was wave 2 suicide ideation. Unadjusted and adjusted estimates are presented. The adjusted estimates were controlled for wave 1 suicidal ideation and other wave 1 covariates: gender, age, ethnicity, marital status, social grade, employment status, health problems, risky alcohol consumption and PGSI status. Analyses were weighted using the wave 2 longitudinal weight, which accounted for initial non-response at wave 1 and attrition between waves. The exploratory analyses for suicide attempts are reported in Supplementary Table 3. Regression models were conducted using Statistical Software version 14 for Windows (StataCorp, College Station, Texas, USA; https://www.stata.com/).

### Role of the funding source

This project was funded by Gambling Research Exchange Ontario (GREO). GREO had no role in the study design, data collection, data analysis, data interpretation or writing of the paper. The corresponding author had full access to all study data and took final responsibility for submission of the publication.

## Results

**Fig. 1:**
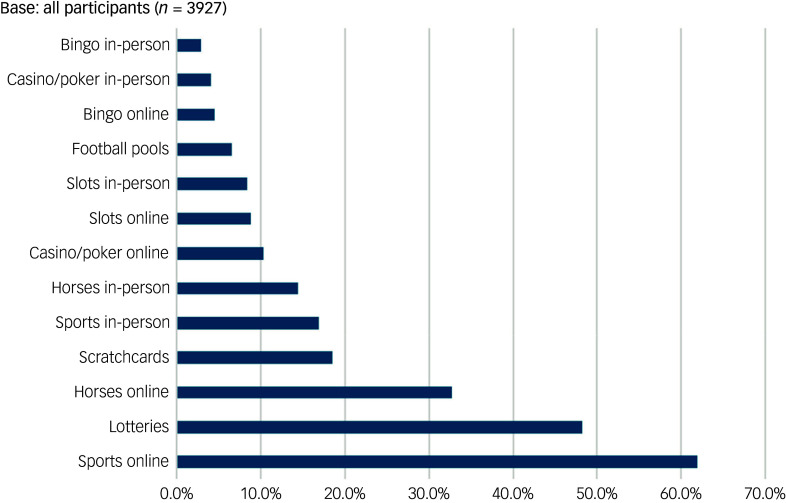
Proportion of participants taking part in each activity at least once per fortnight (before the COVID-19 pandemic).

Overall, 3129 of 3927 (79.7%, weighted) participants at wave 1 were men, 3750 of 3927 (95.3%, weighted) were White/White British and 1640 of 3927 (41.6%, weighted) were aged 35–54 years. The most common gambling activities undertaken regularly (fortnightly or more) were betting on sports online, followed by lotteries, betting on horses online and scratchcards (Fig. 1). Fewer than one in ten participants took part in non-sports betting activities such as casino games or slots, and 562 of 3927 participants (16.3%, weighted) had a PGSI score of 3 or more. At wave 1, 450 of 3927 participants (13.2%, weighted) reported suicidal ideation in the past 3 months, and 53 (1.7%, weighted) reported a suicide attempt in the past 3 months.

### Typologies of people based on regular gambling activity

Results from the LCA and model fit statistics are provided in Supplementary Table 1. Fit statistics indicated that the five-class solution was the optimal model (see Supplementary Material for details), with each group comprising people who engaged in similar patterns of regular gambling activity (Table 1).

**Table 1:** Gambling activities of the subgroups within the five-class solution

Class	Gambling group	Gambling activities
1	In-person: sports and horses	Moderate probability of in-person horses and sports gambling; low probability of all other gambling activities (except lotteries)
2	Online: sports and horses	Moderate probability of horses and sports online gambling; low probability of all other gambling activities (except lotteries)
3	Online and in-person: sports and horses	High probability of horses and sports gambling, both online and in-person; low probability of other gambling activities (except lotteries)
4	Online: all activities	Moderate to high probability of gambling on all online activities (including horses, sports, poker, slots, bingo); low probability of all land-based gambling (except lotteries)
5	Online and in-person: all activities	Moderate to high probability of gambling on all activities, online and in-person (including horses, sports, poker, bingo, slots, football pools, scratchcards)

The groups were differentiated by whether participants bet on: (a) things other than sports/horses; or (b) betting online, in-person or both. The first three classes represented those who tended to regularly bet on sports and/or horses but not other activities. The difference between these three groups was how people placed their bets: in-person only (class 1, in-person: sports and horses, *n* = 548; 12.0%, weighted); online only (class 2, online: sports and horses, *n* = 2756, 71.6%, weighted) or both online and in-person (class 3, online and in-person: sports and horses, *n* = 153, 3.5%, weighted). The remaining two groups represented those who regularly bet on sports and/or horses as well as a range of other activities. These two groups were also differentiated by how they gambled. Participants in class 4 (online, all activities; *n* = 272, 7.2%, weighted) had low probabilities of betting regularly on most in-person activities but had high probabilities of gambling on most online activities, including online casino and slots as well as sports betting. Those in class 5 (online and in-person: all activities; *n* = 198, 5.6%, weighted) regularly gambled on a wide range of activities both online and offline. They were the only group to have high probabilities of gambling in-person on slot machines (Supplementary Fig. 2). All groups had moderate to high probability of playing lotteries; this did not tend to distinguish between the groups.

### Differences in wave 1 characteristics by LCA group

Table 2 outlines the differences among classes in terms of demographics, gambling activities, well-being and suicidality at wave 1. Chi-squared tests indicated that the classes all differed with respect to these variables. Regarding demographics, classes 1 and 3, who only regularly bet on sports but did so in-person, had the oldest age profile (those aged 55 and over), whereas those who gambled online and in-person on all activities (class 5) were the youngest. Class 5 had the highest proportions of those who did not identify as White/White British (17.8%, weighted, Asian, Black or other). Differences between groups by socioeconomic status were evident. Those who gambled online and in-person on all activities (class 5) and those who gambled online on sports and/or horses only (class 2) were more likely to have higher educational attainment, lower levels of unemployment and higher social grade classifications than other groups.

**Table 2:** Differences between the classes: demographic characteristics, gambling risk variables, well-being and suicidality of the five latent classes at wave 1 (*n* = 3927)

Characteristic	Class 1 In-person: sports and horses (*n* = 548), *n* (%)	Class 2 Online: sports and horses (*n* = 2756), *n* (%)	Class 3 Online and in-person: sports and horses (*n* = 153), *n* (%)	Class 4 Online: all activities (*n* = 272), *n* (%)	Class 5 Online and in-person: all activities (*n* = 198), *n* (%)	Total sample (*n* = 3927), *n* (%)	*χ* ^2^	*P*
Demographics								
Age							275.902	<0.001
18–34 years	21 (6.5)	463 (27.8)	10 (11.2)	42 (26.0)	65 (43.2)	601 (25.4)		
35–54 years	159 (32.8)	1192 (42.0)	63 (43.7)	140 (49.3)	95 (43.1)	1649 (41.6)		
55+ years	368 (60.7)	1101 (30.1)	80 (45.1)	90 (24.6)	38 (13.7)	1677 (33.0)		
Gender (male)	444 (81.0)	2171 (78.8)	145 (94.8)	213 (78.3)	156 (78.8)	3129 (79.7)	23.942	<0.001
Ethnicity (White)	531 (96.7)	2647 (95.9)	145 (94.9)	265 (97.5)	162 (81.0)	3750 (95.3)	112.694	<0.001
Employment							159.118	<0.001
Employed	268 (52.4)	1957 (74.4)	99 (66.7)	194 (73.0)	174 (87.6)	2692 (72.1)		
Retired	194 (31.8)	534 (14.5)	31 (17.1)	35 (9.5)	8 (3.9)	802 (15.9)		
Unemployed/other	86 (15.8)	265 (11.1)	23 (16.2)	43 (17.5)	16 (8.5)	433 (12.2)		
Education (higher)	177 (43.6)	1499 (72.6)	45 (41.1)	129 (66.3)	110 (70.7)	1960 (67.6)	149.627	<0.001
Social grade (lower)	265 (48.5)	778 (29.2)	61 (41.8)	87 (33.4)	52 (28.3)	1234 (32.2)	75.841	<0.001
Home-owner	319 (60.3)	1777 (64.8)	94 (63.6)	159 (55.4)	108 (57.7)	2457 (63.8)	17.989	<0.001
Married or cohabiting	375 (68.1)	1830 (63.7)	120 (65.8)	155 (53.3)	139 (68.2)	2601 (63.8)	19.211	<0.001
Health problems	185 (33.2)	551 (19.2)	42 (28.6)	61 (22.5)	83 (44.4)	922 (22.9)	109.343	<0.001
Gambling variables								
PGSI^a^ (moderate/high-risk gambler)	60 (11.6)	247 (10.4)	31 (22.0)	101 (41.6)	123 (65.8)	562 (16.3)	604.423	<0.001
Gambling harm (yes)	21 (4.1)	59 (2.7)	13 (8.9)	39 (13.7)	85 (45.1)	217 (6.2)	656.604	<0.001
Time spent (daily)							1145.928	<0.001
Less than 30 min	163 (29.6)	1168 (46.1)	0 (0.0)	1 (0.4)	0 (0.0)	1448 (36.6)		
30 min to 3 h	285 (51.4)	1168 (42.4)	79 (50.6)	109 (41.5)	26 (12.5)	1667 (42.0)		
3 h+	100 (19.0)	304 (11.5)	74 (49.4)	162 (58.1)	172 (87.5)	812 (21.4)		
Amount spent							491.012	<0.001
£0–50	179 (32.7)	1196 (45.2)	12 (8.6)	37 (15.2)	22 (11.5)	1446 (38.4)		
£51–250	245 (45.6)	1133 (40.8)	68 (44.3)	96 (35.3)	68 (34.0)	1610 (40.7)		
£251+	548 (21.7)	426 (14.0)	73 (47.1)	272 (49.5)	198 (54.5)	870 (21.0)		
Number of activities undertaken fortnightly or more^b^							*F* = 1891.22; d.f. 4.	<0.001
Mean (s.d.)	2.7 (1.1)	1.6 (1.0)	5.0 (1.1)	4.3 91.2)	7.7 (2.2)	2.4 (2.0)		
Alcohol use (AUDIT-C)								
Increasing and/or higher-risk drinking	314 (59.0)	1523 (57.1)	105 (68.9)	155 (59.0)	130 (68.5)	2227 (58.5)	16.53521	0.002
Well-being^c^							54.352	<0.001
Low	176 (33.8)	944 (36.6)	59 (39.6)	122 (50.3)	92 (47.1)	1393 (38.0)		
Moderate	266 (48.2)	1429 (51.1)	62 (39.2)	120 (41.5)	76 (38.6)	1953 (48.9)		
High	100 (18.0)	372 (12.3)	32 (21.2)	27 (8.2)	30 (14.3)	561 (13.1)		
Suicidality (3 months)								
Suicidal ideation	49 (10.5)	306 (12.8)	16 (11.5)	32 (13.0)	47 (27.5)	450 (13.2)	38.834	<0.001
Suicide attempt	5 (1.1)	9 (0.6)	2 (1.5)	6 (2.4)	31 (18.5)	53 (1.7)	337.956	<0.001

PGSI, Problem Gambling Severity Index; AUDIT-C, Alcohol Use Disorders Identification Test Consumption.

a. Has gambling/betting caused any difficulties with your finances, relationships or mental/physical health?

b. Variation in the means tested using analysis of variance.

c. Short Warwick-Edinburgh Mental Wellbeing Scale: low, 7–20; moderate, 21–27; high, 28+.

Patterns of gambling behaviours varied by group. Those who gambled online and in-person on all activities (class 5) spent greater amounts of time and money gambling than other groups. In this group, 123 of 198 participants (65.8%, weighted) had a PGSI score of 3 or more, compared with, 247 of 2756 participants (10.4%, weighted, *P* < 0.001) who bet online on sports and/or horses only.

Both suicidal ideation and suicide attempts measured at wave 1 varied by LCA group, with rates of both being significantly higher among those who gambled online and in-person on all activities (class 5) than other groups: class 5 suicidal ideation *n* = 47/198 (27.5%, weighted) *v*. 49/548 (10.5%, weighted) for in-person sports/horse betting (*P* > 0.001); class 5 suicide attempts *n* = 31/198 (18.5%, weighted) *v*. 9/2756 (0.6%, weighted) for class 2 online: sports/horse only (*P* < 0.001).

### Longitudinal analysis using LCA groups

Table 3 shows bivariate associations between LCA groups and suicidal intention at wave 2. Binary logistic regression was conducted for suicidal ideation at wave 2, with LCA class at wave 1 as the exposure variable and the reference category changed to compare risk for each group. The odds ratios for each binary logistic regression for the LCA groups are reported in Table 4 (see Supplementary Table 4 for results for all covariates and Supplementary Table 3 for results of logistic regression models with suicide attempts as the outcome).

**Table 3:** Suicidal ideation and suicide attempts for each latent class at wave 2

Suicidality	Class 1 In-person: sports and horses	Class 2 Online: sports and horses	Class 3 Online and in-person: sports and horses	Class 4 Online: all activities	Class 5 Online and in-person: all activities
	*n* (%)	*n* (%)	*n* (%)	*n* (%)	*n* (%)
Wave 2	448	2184	123	205	133
Suicidal ideation	29 (9.2)	234 (13.6)	9 (9.4)	26 (11.4)	34 (35.4)
Suicide attempt	1 (0.4)	9 (0.4)	0 (0)	3 (1.2)	24 (27.8)

**Table 4:** Adjusted binary logistic regression analyses using latent classes to predict suicidal ideation at wave 2 (*n* = 3093) with different reference groups

Latent class analysis group	Wave 2: suicidal ideation odds ratios [95% CI]	
	Unadjusted	Adjusted^a^
Class 1 reference		
Class 2	1.56 [0.97–2.50]	1.70 [0.90–3.19]
Class 3	1.03 [0.43–2.46]	0.79 [0.252–2.47]
Class 4	1.27 [0.67–10.63]	1.04 [0.46–2.37]
Class 5	5.43 [2.77–10.63]***	3.56 [1.17–10.84]^*^
Class 2 reference		
Class 3	0.66 [0.30–1.43]	0.46 [0.17–1.29]
Class 4	0.82 [0.50–1.34]	0.61 [0.33–1.15]
Class 5	3.48 [2.02–6.00]***	2.10 [0.80–5.48]
Class 3 reference		
Class 4	1.24 [0.51–3.02]	1.32 [0.427–4.072]
Class 5	5.29 [2.12–13.22]***	4.51 [1.21–16.84]^*^
Class 4 reference		
Class 5	4.27 [2.13–8.52]***	3.42 [1.18–9.89]^*^

a Analyses adjusted for: wave 1 suicidal ideation or suicide attempt (dependent on outcome), gender, age, ethnicity, social status, marital status, health problems, Alcohol Use Disorders Identification Test for problem drinking, employment status, Problem Gambling Severity Index.

* *P* < 0.05, **P < 0.01, ***P < 0.001.

### Wave 2 suicidal ideation

In the adjusted binary logistic regressions, when suicidal ideation at wave 2 was the outcome variable, individuals who gambled online and in-person on all activities (class 5) had higher levels of suicidal ideation compared with: those who bet in-person on sports and/or horses (class 1) (odds ratio 3.56; 95% CI: 1.17–10.84); those who gambled online and in-person on sports and/or horses (class 3) (odds ratio 4.51; 95% CI: 1.21–16.84); and those who gambled online on all activities (class 4) (odds ratio 3.42, 95% CI: 1.18–9.89).

Supplementary analysis of adjusted binary logistic regression for suicide attempts suggested that individuals who gambled online and in-person on all activities (class 5) had a higher risk of suicide attempt(s) than those who: gambled in-person on sports and/or horses (class 1) (odds ratio 18.78, 95% CI: 2.03–173.53); gambled online on sports and/or horses (class 2) (odds ratio 23.36; 95% CI: 8.25–66.09); and those who gambled online only on all activities (class 4) (odds ratio 21.0; 95% CI: 3.93–112.16). However, base sizes for these analysis were small, and the analyses were underpowered.

## Discussion

Across the whole sample, all participants reported rates of suicidal ideation and attempts higher than national estimates, consistent with evidence of increased risk among people who regularly engage in non-lottery-based gambling. To our knowledge, this is the first study to examine the relationship between different types of gambling activity and subsequent suicidal ideation longitudinally. Among our sample of regular sports bettors, people who gambled both online and in-person on all activities (class 5) were more likely to report suicidal ideation 3 months later than most other groups in this sample of people already at elevated risk. The main factors distinguishing participants in class 5, representing 5.6% of the sample, from the other groups was their higher regular engagement across multiple activities and their high propensity to gamble regularly on electronic gambling machines (EGMs). Although we need to be cautious when interpreting the supplementary analyses for suicide attempts, the pattern of findings was similar to those for suicidal ideation.

These findings extend existing knowledge about factors associated with highest risk of harms. Cross-sectional evidence has shown that people who engage in a wider range of gambling activities are more likely to experience gambling harms.^23–25^ In this study, those who gambled both online and in-person across multiple activities had a higher propensity to experience gambling harms, as evidenced by the elevated proportions in this group with PGSI scores of 3 or more. Crucially, adjusted regression analyses showed that membership of this group was strongly associated with subsequent suicidal ideation, even when PGSI score was taken into account. Thus, factors other than elevated PGSI scores underlie this observed association. Evidence shows that engagement in fast-paced, continuous forms of gambling, such as EGMs and their online counterparts, is among of the strongest risk factors for harms.^26^ It is thus notable that a high propensity to use EGMs differentiated class 5 from other groups, suggesting that both wider gambling engagement and engagement in specific gambling formats may underlie this association.

These data were collected during the COVID-19 pandemic, which may have influenced the results. Our analysis took into account multiple factors which may have been affected by the pandemic (including health, alcohol and employment) but was not able to adjust for all COVID-related experiences. Thus, it remains possible that other experiences during this time period may underlie these observations. Further limitations should be recognised. From a sampling perspective, this study used a non-probability sample, with attendant issues for generalisability. However, people who gamble regularly can be a seldom-heard group, and online panels, as used in this study, allow identification of the target population. Although non-probability panels are not advised for prevalence estimates, they perform somewhat better when focusing on the relationship between variables, as this study did.^27^ Sample sizes for suicide attempts were small, especially when examined by LCA group, and caution should be taken with these findings. Regarding measurement limitations, all estimates were self-reported and susceptible to recall error or social desirability bias, although the online administration of the survey should have helped to minimise the latter.

These results have important implications for identification and management of gambling harms. To date, research has focused on problematic gambling severity and suicidality. The results presented here suggest that the range and type of gambling formats may be more important indicators for identification of those at greatest risk of subsequent suicidal ideation in a cohort already known to experience elevated risk. It will be key to consider the psychological profiles of those who engage in continuous gambling formats such as EGMs to determine whether they report higher levels of shame, defeat and entrapment, recognised risk factors for suicide.^28^ It would also be useful to explore the extent to which individuals in the different classes are characterised by distinct motivational systems associated with one’s sensitivity to punishment (behavioural inhibition system) and reward (behavioural activation system).^29^ In addition, future research could explore the extent to which suicidal ideation varies according to different volitional factors for gambling-related suicidality, for example, mobile phone gambling giving instant and constant access to gambling.

These findings have clear implications for the implementation of the new National Institute of Health and Care Excellence guidelines on gambling-related harms.^7^ Currently, guidelines focus on asking people whether they are worried about their own or someone else’s gambling as an introduction to the topic. On the basis of this study, discerning follow-up questions focused on gambling behaviours, such as whether a person gambles regularly on a range of activities, including online and offline slots, may elicit further insight to help healthcare professionals to identify and manage heightened risk of suicidal ideation. In short, gambling should be considered routinely in psychosocial assessments across clinical settings, not just primary care, and incorporated into suicide prevention campaigns.

These findings also have further implications for the regulation of gambling. The Gambling Commission, the industry regulator, recently announced that it would ban mixed-product cross-promotions, essentially prohibiting an incentive or bonus offer that requires customers to participate in more than one type of gambling product. The rationale given for the change was the recognised additional risk that taking part in more than one type of gambling generates. Our findings support this policy action and suggest that further regulatory action to reduce operator cross-selling between gambling formats could be considered.

Longitudinally, a particular profile of gambling activity was found to more strongly predict subsequent suicidal ideation than other profiles. The supplementary analyses also pointed to a similar relationship with suicide attempts. These relationships need further investigation and replication with data not confounded by the COVID pandemic. However, the findings suggest that those who are most engaged with a range of gambling formats, especially both online and offline slots, should be viewed as being at heightened risk for subsequent suicidal ideation even when compared with others at elevated risk.

## Supporting information

Wardle et al. supplementary material Wardle et al. supplementary material

## Data Availability

Data are available at https://osf.io/u3wkx/files/osfstorage.

## Supplementary material

The supplementary material is available online at https://doi.org/10.1192/bjo.2025.10935
