# Supplementary material for: Suicidal ideation among people with different gambling behaviour profiles: analysis of a longitudinal survey of people who gamble regularly in the UK
Source: BJPsych Open. 2026 Jan 15;12(1):e38. doi: 10.1192/bjo.2025.10935 (PMC12835708; doi:10.1192/bjo.2025.10935)
Supplement: Wardle et al. supplementary material [file S2056472425109356sup001.docx]

**Supplementary materials**

**Table S1. Combinations of gambling activities for analysis**

| **Individual Gambling Activity** | **Grouped gambling activity category** |
| --- | --- |
| Online Horse race betting | Online horse race betting |
| Online sports betting | Online sports betting |
| Online betting on esports | Online sports betting |
| Online betting on virtual sports | Online sports betting |
| Online poker | Online casino/Poker |
| Online casino games | Online casino/Poker |
| Online slots | Online slots |
| Online bingo | Online bingo |
| Horse race betting in person | Horse race betting in person |
| Sports betting in person | Sports betting in person |
| Betting on esports in person | Sports betting in person |
| Betting on virtual sports in person | Sports betting in person |
| Table games at a casino | Casino in person |
| Poker at a club | Casino in person |
| Slot/Fruit machines | Slots in person |
| Machines at a bookmakers | Slots in person |
| Bingo at a hall/club | Bingo in person |
| Lotteries | Lotteries |
| Scratchcards | Scratchcards |
| Football pools | Football pools |

**Choice of LCA results:**

To determine the optimum LCA solution we examined model fit statistics and theoretical interpretability. Regarding model fit statistics, lower Bayesian information criterion (BIC) and the Akaike information criteria (AIC) suggest better model fit (Nylund, Asparouhov, & Muthén, 2007). Other fit statistics can be used, such as the bootstrap likelihood ratio test (B-LRT; McLachlan & Peel, 2000) which tests whether the more complex model (e.g., 5-class) fits the data significantly better than the simpler model (e.g., 4-class). A further test statistic is entropy, which indicates how accurately the model defines the classes; a value closer to 1 suggests better class separation, and anything above 0.8 is considered good (Muthén & Muthén, 2007). To further determine whether individuals had been assigned into the best fitting class, the average latent class posterior probability (i.e., the average probability of the class model accurately predicting class membership) can also be assessed (B. Muthén & Muthén, 2000). Latent probabilities are displayed in a matrix with diagonals representing the average probability of a person being assigned to a class given his or her scores on the indicator variables, and higher diagonal values (i.e., closer to 1.0) are desirable. Finally, the number or percentage of the sample assigned to each group can be assessed, with a group less than 5% suggested to be too low, although more recent literature suggests that if the group has a theoretical basis then there is no issue (Weller et al., 2020).

Fit statistics for the 2-class up to the 6-class model are shown in Figure S1. The BICs and AICs for the models decrease as the number of classes increased and tapered around the 4 to 5 class model. Further, across the models the bootstrap likelihood ratio test (BLRT) found that the more complex model was a significantly better fit than the simpler. The 6-class model was rejected as too many groups had less than 5% of the sample and it made less theoretical sense. On comparing the 4 and 5-class solutions (see Table S2), the classes within both had an average latent class posterior probability above 0.8, although both included some small groups (~5%), and the 5-class solution included a group with around 4% of the sample. The 5-class solution had an entropy that was above 0.8, whereas the 4-class solution was 0.76. As the fit statistics gave a relatively unclear indication to which model had the best fit, the solutions were assessed for theoretical interpretation, and consensus agreed that the 5-class solution was a better reflection of the data and gambling behaviours.

**Figure S1. Plotting the Bayesian information criterion (BIC) and the Akaike information criteria (AIC) to include model fit.**

**Table S2. Comparison of the 4- and 5-class solution on the average latent class posterior probability and size of each class.**

|  | 4-class solution | | 5-class solution | |
| --- | --- | --- | --- | --- |
|  | Latent class probability | Group size, n (%) | Latent class probability | Group size, n (%) |
| Class 1 | 0.883 | 2641 (67.3) | 0.876 | 548 (14.0) |
| Class 2 | 0.914 | 224 (5.7) | 0.809 | 2756 (70.2) |
| Class 3 | 0.883 | 802 (20.4) | 0.900 | 153 (3.9) |
| Class 4 | 0.805 | 260 (6.6) | 0.899 | 272 (6.9) |
| Class 5 | - | - | 0.851 | 198 (5.0) |

**Table S3: Adjusted binary logistic regression analyses using latent classes to predict suicidal ideation and suicide attempt at wave 2 (n=3093) with different reference groups.**

|  | **Wave 2: Odds Ratios of Suicide Attempts [95% CI]** | |
| --- | --- | --- |
|  | Unadjusted | Adjusted |
| **Class 1 reference** |  |  |
| Class 2 | 1.04 [0.12-8.56] | 0.80 [0.11-6.12] |
| Class 3 |  | - |
| Class 4 | 2.83 [0.29-27.66] | 0.89 [0.06-12.89] |
| Class 5 | 93.23 [12.06-720.48]*** | 18.78 [2.03-173.53]* |
| **Class 2 reference** |  |  |
| Class 3 | - | - |
| Class 4 | 2.73 [0.68-11] | 1.11 [0.16-7.53] |
| Class 5 | 89.99 [34.35-235.76]*** | 23.36 [8.25-66.09]*** |
| **Class 3 reference** |  |  |
| Class 4 | - | - |
| Class 5 | - | - |
| **Class 4 reference** |  |  |
| Class 5 | 32.91 [9.08-119.2]*** | 21.00 [3.93-112.16]*** |

^+^Analyses adjusted for: wave 1 suicidal ideation or suicide attempt (dependent on outcome), Sex, Age, Ethnicity, Social status, Marital status, Health problems, AUDIT for problem drinking, Employment status, PGSI for problem gambling. * p <0.05, ** p<0.01, ***p<0.001

Class 3 omitted from suicidal attempts analysis as there were no observations.

**Table S4. Binary logistic regression using latent classes to predict suicidal ideation and suicide attempt at wave 2 (n=3093)**

|  | Wave 2 OR [95% CI] | |
| --- | --- | --- |
|  | Suicidal ideation | Suicide attempt |
| Class 1^a^ | 1 |  |
| Class 2 | 1.697 [0.904-3.185] | 0.804 [0.106-6.115] |
| Class 3 | 0.788 [0.252-2.466] | - |
| Class 4 | 1.04 [0.456-2.374] | 0.895 [0.062-12.891] |
| Class 5 | 3.558 [1.168-10.840]* | 18.784 [2.033-173.534]* |
| Wave 1 Suicidal Ideation or attempt^b^ | 27.305 [17.898-41.657]*** | 10.295 [3.939-26.908]*** |
| Sex^c^ | 0.626 [0.382-1.027] | 0.334 [0.134-0.832]* |
| Age: 18-34 yrs^d^ | 2.615 [1.338- 5.108]** | 4.821 [0.755-30.764] |
| Age: 35-54 yrs | 2.007 [1.142-3.525]* | 8.529 [1.614-45.087]* |
| Ethnicity^e^ | 1.437 [0.359-5.755] | 0.376 [0.097-1.456] |
| Social grade^f^ | 1.344 [0.796-2.269] | 5.063 [1.084-23.657]* |
| Marital status^g^ | 0.835 [0.553-1.261] | 0.581 [0.203-1.666] |
| Health problem^h^ | 0.332 [0.214-0.516]*** | 0.147 [0.050-0.431]*** |
| Alcohol use^i^ | 1.088 [0.703-1.683] | 0.963 [0.326-2.847] |
| Employed^k^ | 0.67 [0.340-1.321] | 1.189 [0.198-7.147] |
| Retired | 0.913 [0.328-2.546] | 9.419 [1.110-79.910]* |
| Moderate/high risk gambler^l^ | 0.864 [0.509-1.465] | 2.37 [0.717-7.839] |

Reference group: ^a^ Class 1, ^b^ No suicidal ideation/attempt, ^c^ Female, ^d^ 55+ yrs, ^e^ Asian or Black or Other, ^f^ Lower (C2-E), ^g^ Not married/co-habiting, ^h^ Has health problems, ^i^ AUDIT lower risk drinking, ^k^ Unemployed/Sick, ^l^ Non problem/low risk gambler.

– No OR generated as no cases.

**Figure S2. Estimated probability of individuals in each class taking part in the gambling activities at least once a fortnight**

Note: HORSE-O = horses online, SPORTS-O = sports online, CASINO/POKER-O = casino games or poker online, SLOT-O = slots online, BINGO-O = bingo online, HORSE-IP = horses in-person, SPORTS-IP = sports in-person, CASINO/POKER-IP = casino or poker in-person, SLOTS-IP = slots in-person, BINGO-IP = bingo in-person, POOLS = football pools, LOTTERY = national lottery, SCRATCH = scratch cards.
